# Using neuroimaging to identify sex differences in adults with sports-related concussion: a systematic review

**DOI:** 10.1007/s11682-025-00970-6

**Published:** 2025-01-24

**Authors:** Harry Macleod, Clare L. Smith, Robin Laycock

**Affiliations:** Royal Melbourne Institute of Technology, 124 La Trobe Street, Melbourne, VIC 3000 Australia

**Keywords:** Concussion, mTBI, Neuroimaging, Sex differences, Sports-related concussion

## Abstract

Concussion is a common injury in sports that causes neurological damage, leading to memory loss and difficulty concentrating. Insufficient recovery time may result in significant long-term harm to individuals. Several neuroimaging techniques have been used to understand the pathophysiological changes following concussion, and how long individuals need to recover before returning to play. Despite the progress in neuroimaging concussion research, few studies have considered whether females sustain different effects on the brain and how recovery from concussion might differ from males. Thus, we conducted a systematic review of the existing literature to highlight sex differences in concussion with neuroimaging techniques. By searching four different databases, studies were selected if they used a neuroimaging technique to examine sex differences following concussion in athletes over the age of 18. After screening 2295 studies from an initial search, 15 were found to match the selection criteria. Nine papers established some difference between males and females, however many of these studies were not designed to specifically examine sex differences, and hence conclusions in this regard are somewhat limited. A further common limitation among these papers was a lack of whole brain scans, instead relying on regions of interest analyses, which reduces the ability to compare studies effectively. The current systematic review has highlighted the need for future studies to specifically consider whether, and how sex influences the impact and trajectory of brain recovery from concussion. This can then help to inform suitable amendments to current concussion return-to-play protocols for male and female athletes.

**Supplementary Information:**

The online version contains supplementary material available at 10.1007/s11682-025-00970-6.

## Introduction

Concussion is a form of mild traumatic brain injury (mTBI), caused by mechanical trauma disrupting brain function (Menon et al., 2010). It is estimated that 3.8 million concussions occur in the US each year (Harmon et al., 2019). More awareness has been raised about the long-term impacts of concussion, as athletes from a range of contact sports have begun to present with brain damage following multiple concussions (Mez et al., 2020). In the sporting context, concussion protocols have now become a priority, as re-injury prior to full recovery can cause serious health consequences (Vagnozzi et al., 2008). The Sport Concussion Assessment Tool (SCAT6) is commonly used as part of protocols in sport to ensure people are returned to play safely (Patricios et al., 2023). These types of tests involve a brief assessment of self-observed and reported symptoms, also assessing orientation, memory, and balance. However, they can provide variable responses based on gender and existing diagnoses (e.g., ADHD), and may not be reliable for tracking recovery beyond a few days (Echemendia et al., 2017). Reliance on subjective reporting fails to accurately identify the neurological changes following concussion, as well as when these changes have recovered (Orr et al., 2016). Neuroimaging research has demonstrated a clear representation of neurological abnormalities after symptoms have subsided, providing a more accurate representation of how long recovery can take (Chen et al., 2004).

Concussion studies have heavily focused on male athletes and their recovery (D’Lauro et al., 2022), despite evidence suggesting differences in recovery time between males and females following concussion (Gallagher et al., 2018). Whilst female contact sport is still growing, identifying any differences in their concussion recovery processes compared to male athletes would be beneficial in creating appropriate female concussion protocols. Neuroimaging could be particularly useful in identifying such differences, as it can highlight the micro-scale changes occurring as athletes recover from concussion (Gonzalez et al., 2021; Churchill, 2021). Various Magnetic Resonance Imaging (MRI) techniques such as functional MRI (fMRI), Magnetic Resonance Spectroscopy (MRS) and Diffusion Tensor Imaging (DTI) could improve concussion protocols and minimize later-life neuro-behavioural complications.

### Concussion: neurobiology and sporting protocols

A concussion involves changes in brain cell membranes and axon structure, which results in metabolic responses to heal the damage (Giza & Hovda, 2014). During this time, inflammation increases microglial activation (Giza & Prins, 2006), which leads to symptoms of memory loss, reduced attention span and mood changes. The brain is most vulnerable during this time, and long-term damage can occur if there is another concussion prior to full recovery (Vagnozzi et al., 2008). Ultimately, repeated concussive events can result in issues like chronic traumatic encephalopathy (CTE), which causes brain volume loss often resulting in serious mental and physical decline (Gavett et al., 2011).

Therefore, concussion protocols should be clear and provide ample recovery time for players to avoid long-term effects. However, in current popular sports, the concussion guidelines are inconsistent between sports. For example, the men’s and women’s Australian rules Football League (AFL and AFLW) enforces a 12-day return to play policy following a concussion event (AFL, 2023), the English Football Association also enforces a 12 day policy but this can be reduced to 7 days if exceptional criteria are met (Football Association, 2023), while the NFL in America provide no required minimum date to return, basing the decision exclusively on symptom testing (NFL Head, Neck and Spine Committee, 2022).

Another issue with the current protocols is a one size fits all approach, and fails to consider the individual needs for adequate recovery, concussion histories and demographics of those affected (Starling et al., 2022; Shultz et al., 2013). The current protocols lack longitudinal testing, and by assuming a universal recovery time could be dangerous for some athletes’ long-term health.

To remedy the issue, further concussion research is required to examine how males and females may differ in recovery. While other factors (e.g., demographic variables, mechanism of injury) also contribute to the pathophysiology of the injury (see for example Joyce et al., 2022) we focus on sex as a potentially important factor. Neuroimaging has been used in research to highlight sex differences (Covassin & Elbin, 2011), allowing the observation of microscale changes that occur following concussion and contribute to improved understanding of individual differences in time to recovery.

### Neuroimaging techniques that offer valuable concussion analysis

When identifying appropriate neuroimaging to assess sex differences, it is important that adequate biomarkers are utilised that are capable of showing brain activity changes after concussion (Kodiweera et al., 2016). Key technologies which have been suggested to be effective in this analysis includes fMRI, DTI and MRS (Giza & Hovda, 2014). Each of these techniques have their own strengths, but when combined provide a well-rounded view of the biological reactions following sports-related concussion.

FMRI uses a strong magnetic field to change proton spin of hydrogen molecules, an important element in haemoglobin (Kim & Gean, 2011): this can help identify regional blood flow changes in the brain (the Blood Oxygenation Level Dependent, BOLD response), indicative of neural activity change (Guell et al., 2020). This is important in identifying neural areas of weakness following concussion and scans at different times post-injury can highlight longitudinal effects. In addition, fMRI has identified disruption to functional connectivity in the prefrontal cortex, related to working memory performance in patients, months after concussion recovery (Chen et al., 2004). Functional connectivity measures the strength of synchronous activity patterns between two spatially distinct brain regions, typically while the brain is at rest or engaged in a task (Johnson et al., 2012). These findings suggest functional neuroimaging techniques could be particularly beneficial for identifying sex differences following concussion. This could be enhanced by incorporating other technologies beyond functional deficits that assess how brain axons change following concussion.

Another promising technique is DTI, which measures diffusion in the brain to represent white matter integrity (Assaf & Pasternak, 2008). DTI has highlighted white matter make-up changes whilst tracking athletes throughout their seasons following concussion (Bazarian et al., 2012). This study observed damage to white matter integrity in the temporal lobe and related the injury with poor visual memory performance scores (Collins et al., 2003). Highlighting white matter integrity is crucial because of its role in sending neural signals through the brain (Cubon et al., 2011). Aligning structural findings from DTI with functional findings from fMRI would highlight the impact these axonal changes have on the ability of the brain to function. Mean Apparent Propagator (MAP) MRI is a newer diffusion technique sensitive to microstructural changes in brain tissues (Goeckner et al., 2024). Diffusion techniques like MAP MRI could identify the difference in white matter integrity between males and females following concussion but could be assisted by techniques that can identify metabolic changes.

MRS can identify metabolites in the brain due to resonating differences determined by the target’s chemical structure, when present within a magnetic field (Giza & Hovda, 2014). Changes in neurometabolites like N-acetylaspartate (NAA) levels can give a comprehensive reflection of neuronal density and thus the concussion’s structural impact on the brain (Veeramuthu et al., 2018). Previous studies of female concussed athletes have found significant metabolic concentration differences of NAA compared to female control athletes 6 months after concussion, despite these athletes not presenting any symptoms (Chamard et al., 2014). Using MRS during the recovery period can extend on structural and functional findings from various other techniques and clearly identify how different metabolites contribute to brain recovery.

Different forms of neuroimaging have various strengths in diagnosing concussion, with each technology identifying different changes in the brain. Analysing these findings that focus on different biological changes together can help understand each level of the brain that is impacted by concussion. A prevalent problem with many current neuroimaging studies is an inequality of gender representation, with approximately 80% of concussion studies completed on males (D’Lauro et al., 2022). As female participation in sports rises, it is increasingly important to identify any different neurological changes due to concussion when compared to males to ensure the long-term health of female athletes is protected.

### Female athletes

Previous research has found symptom severity and length of recovery following concussion is typically more for females compared to males (Covassin & Elbin, 2011; Merritt et al., 2019; Broshek et al., 2005). This may be attributed to the biological differences in females that make them more susceptible to injury (Australian Institute of Sport, 2023). Additionally, it has been reported that there may be a difference in the reporting of sports-related concussion, as females may be more honest than males, which is more suggestive of a socially shaped gender difference (Dick, 2009). Despite this, many sporting bodies assume the same concussion recovery period for both males and females. However, as pathophysiology may persist past symptom recovery (Churchill et al., 2021b), identifying how females might physiologically react to concussion differently to males is important in creating more suitable concussion guidelines.

Another reason why males and females might differ in concussion response and recovery relates to sex hormones. As females experience stronger endocrinological fluctuations due to their menstrual cycle, and hormones like progesterone play a neuroprotective role, females can respond to concussion differently based on where they are in their cycle (Gallagher et al., 2018). Indeed, studies have found that out of four possible phases, 50% of females sustain a concussion during their late luteal phase, when both estrogen and progesterone are decreasing (La Fountaine et al., 2019). Unlike males who do not experience fluctuating periods of sex hormones, consideration of these menstrual changes when accounting for concussion recovery is vital in understanding how females recover differently, not only from males, but also from each other.

Another potential source for difference in female recovery from concussion lies in how their brain structure changes post-injury as they react differently to physical impact. Female athletes have been demonstrated to require a longer time for metabolites and structural integrity to recover when compared to male athletes, along with significant differences in effects on the motor cortex and corpus callosum (Chamard et al., 2013). Damage to neural integrity in the motor cortex has been related to strain from rotational forces due to the aggressive manner in which sports-related concussions can occur (Sabet et al., 2008). Furthermore, given that females on average have weaker muscle resistance and head-neck mass compared to males, these rotational forces have a larger effect (Tierney et al., 2008).

### Aims

Concussion can have serious long-term effects if not treated appropriately. The current best practice of relying on clinicians to identify cognitive dysfunction and observing symptoms in individuals (i.e., see best practise tools discussed in the 2022 consensus statement on concussion in sport from the 6th International Conference on Concussion in Sport, Patricios et al., 2023) does not acknowledge the micro-scale changes that occur following concussion. Indeed, one of the most widely used tools particularly in a sporting context, the SCAT6 may not have clinical utility after 7 days post injury, rendering it less useful for return to play decisions (Echemendia et al., 2023). These factors may make the athlete vulnerable to further injury if returned to play too quickly (Chamard et al., 2013). While there is still a need for improved understanding of the mechanisms of concussion in general, there is very little research into female athletes and how their sports-related concussion recovery differs from males. Given the effectiveness of using neuroimaging to investigate the biological response to concussion, the present systematic review aims to synthesise what is currently understood regarding sex differences immediately following sports-related concussion and during concussion recovery.

We note that while there has been more focus on sex differences in mTBI (i.e., with a broader focus than sports-related concussion), even here a recent scoping review into TBI has recently argued that the evidence on sex differences is complicated and often contradictory and in need of further research (Gupte et al., 2019). The authors did suggest that women had worse outcomes than males in mild-moderate, but not for moderate-severe TBI, reinforcing the suggestion that severity and potentially mechanism of injury is an important contributor to predicting outcome. By focussing on sports-related concussion in the present review, it is hoped that such a synthesis can help inform gaps in knowledge and ultimately can guide future sports protocols to account for any existent sex differences in their concussion recovery guidelines.

As a result, this study aimed to systematically evaluate the literature for current neuroimaging assessments used to identify different sex responses following sports-related concussion. Due to the exploratory nature of this study, there are no clear hypotheses stated.

## Method

This systematic review was conducted in line with the preferred reporting items for systematic reviews and meta-analyses (PRISMA). A comprehensive search was completed on April 20, 2024, across multiple databases, including Scopus, PubMed, Web of Science and Proquest Central. The search used the following key terms: (1) ‘concussion’ or ‘traumatic brain injury’ or ‘head injury’, (2) ‘female’ or ‘women’ or ‘sex differences’, (3) ‘neuroimaging’ or ‘brain scan’ or ‘MRI’ or ‘DTI’ or ‘MRS’ or ‘radiopharmaceuticals’ or ‘PET’ or ‘SPECT’ and finally (4) ‘athlete’ or ‘sport’. These terms were based on the National Library of Medicine’s vocabulary thesaurus (National Library of Medicine).

### Selection criteria

For inclusion in this review, studies were required to meet the following criteria: published between 2010 and 2023; a sample including both males and females who were 18 years or older; inclusion of a neuroimaging technique; a participant group of active athletes with a concussion diagnosis resulting from a sports-related injury. This ensured the search focused on groups that were alike and easy to compare to highlight the findings in literature observing sex differences following concussion. Given that differences in concussion recovery have been found between paediatric and adult populations (Davis et al., 2017), a focus on adults was deemed necessary to avoid any results being affected by the age of the sample. A Downs and Black checklist was used to identify any risk of bias (Downs & Black, 1998) and is presented in Supplementary Table 1.

### Data extraction

All papers found from the searches were exported to the systematic review management website, Covidence (Covidence systematic review software, 2023). Data was reviewed and extracted in a three-stage process to compare the final studies in a qualitative format. Papers went through a title screening, an abstract and full body screening, followed by a final extraction. For papers to pass the abstract and full body screening, it had to be verified by two different reviewers who were part of the research team, as recommended by PRISMA guidelines. Any conflicts were first discussed between all three reviewers that were involved in the process prior to extraction.

## Results

**Fig. 1:**
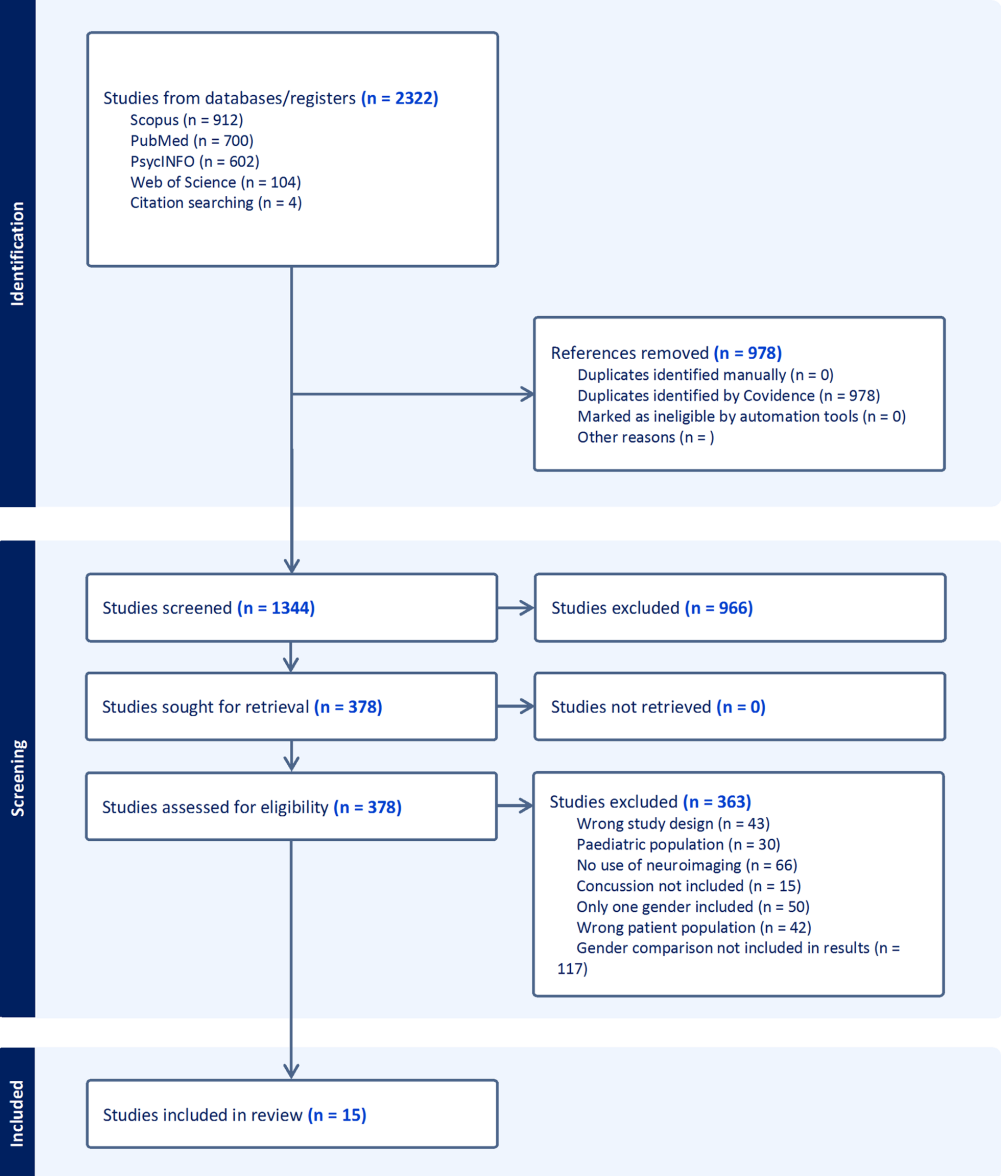
PRISMA flow diagram of the phases of exclusion in this systematic review

A flow diagram of papers that were included and excluded at each stage of PRISMA are shown in Fig. 1.

Table 1 provides a summary of key details regarding the techniques used, the type of evaluation, and the main findings from all studies included in the current systematic review.

**Table 1:** Summary of included studies

First Author (Year)	Population	Neuroimaging Technique	Evaluation Time		Results
			Baseline	Comparison	
Chamard (2012)	America. *n* = 45 (44% female) ice hockey players during their seasons. Male average age: 22.24, Female average age: 20.21.	MRS – focused on corpus callosum	All athletes measured at the start and end of their seasons	If a player had a concussion during the season, they were measured 72 h, 2 weeks, and 2 months post-injury	The NAA/CR ratio did not differ between concussed males and females but did between non-concussed males and females.
Churchill (2017)	Canada. *n* = 26 (54% female) concussed university athletes compared to *n* = 26 (54% female) university athlete controls. Concussed average age: 20, control average age: 20.	Resting state fMRI measured functional connectivity	Control group	Acute phase of injury (1–7 days post-injury)	No significant differences seen in sex according to non-parametric Wilcoxon tests.
Churchill (2020)	Canada. *n* = 61 (51% female) concussed university athletes compared *n* = 167 (52% female) university athlete controls. Concussed average age: 20.4, control average ag: 20.2.	Resting-state fMRI – BOLD temporal dynamics across different time scales (scaling)	Acute phase	Subacute phase (8–14 days post-injury), medical clearance to Return to play (RTP), 1 month post-RTP, 1 year post-RTP	No significant differences found between concussed males and females.
Churchill (2021a)	Canada. *n* = 26 (54% female) concussed university athletes compared to *n* = 167 (52% female) athlete controls. Concussed average age 19.9, control average age 20.2.	Resting-state fMRI scans and structural MRI scans were taken, then compared on local and global network efficiency	Control group	Acute phase, RTP, 1 year post-RTP	No sex effects found.
Churchill (2021b)	Canada. *n* = 61 (51% female) concussed university-level athletes compared to *n* = 167 (52% female) university athlete controls. Male concussed average age: 20.8, female concussed average age: 19.9, male control average age: 20.7, female control average age: 19.8	Arterial Spin Labelling (ASL) and DTI – all data that was relevant was grouped into clusters to be investigated closer, measured based on cerebral blood flow (CBF) change, Fractional Anisotropy (FA) and Mean Diffusion (MD)	Control group	Acute phase, RTP, 1 year post-RTP	No sex difference identified in clinical presentations. Imaging data found that: 1. males had greater reductions in occipital-parietal CBF than female athletes throughout all phases of measurement. 2. Decreased FA found in concussed females compared to males, particularly in the corpus callosum and posterior thalamic radiation. 3. The greatest effects in CBF and MD were found at 1-year post-RTP for all concussed participants
Goeckner (2024)	America. *n* = 56 (23% female) university-level athletes who had played sports in the last 12 months with a concussion occurring six or more months prior to testing compared to *n* = 55 (45% female) university level athletes who had played sports in the last 12 months and had no history of concussion. Concussed average age: 21.5, non-concussed average age: 21.1	MAP-MRI measured diffusion in 10 white matter and eight subcortical grey matter ROIs	Control group of athletes with no concussion history	Any time six or more months after concussion	Lower white matter non-gaussian (NG) mean for concussed females compared to concussed males, particularly in the forceps major and minor, and left cingulate gyrus.
Hamer (2020)	Canada. *n* = 56 (50% female) university level athletes who had experienced a concussion compared to *n* = 66 (50% female) university athlete controls. Concussed male average age: 21.7, concussed female average age: 20.4, control male average age: 19.8, control female average age: 20.1	ASL used to measure CBF changes	Control group	Anytime following a concussion	1. Concussed males had lower CBF relative to controls, whilst concussed females had a higher standard deviation within their group compared to the concussed male group. 2. Females with multiple previous concussions had lower CBF than those with a single concussion. 3. Changes in males were predominantly temporal, which is important for memory.
Jarrett (2016)	Canada. *n* = 11 (55% female) concussed university ice hockey players compared to *n* = 15 (60% female) university student controls. Concussed average age: 21.2, control average age: 22.9	MRI scans to investigate brain volume changes compared to baseline and to controls	All athletes received preseason scans – those who were not concussed during the season were the control group	Athletes with concussion were scanned 72 h following concussion, 2 weeks, and 2 months after concussion	Percent brain volume change found no difference between concussed males and females.
Ly(2022)	America. *n* = 29 (47% female) concussed university athletes compared to *n* = 48 (49% female) university athlete controls. Average age: 20.28	Resting fMRI measured and combined into eight resting state networks, as well as DTI	Control group	72 h following injury	1. Decreased functional connectivity between frontoparietal/default mode and visual networks in concussed compared with non-concussed males, but no difference between concussed and non-concussed females 2. No sex differences in DTI measures
Panchal (2018)	Canada. *n* = 33 (50% female) university ice hockey athletes. Male average age: 22.0, female average age: 20.2	MRS particularly focused on the corpus callosum, highlighting NAA, Choline and Glutamate	Pre- season testing taken for all players	Post-season testing taken for all players (investigating effect of repetitive head impacts, controlling for incidence of concussion)	Both male and female athletes had a persistent decrease in NAA post-season. A nonsignificant interaction between time and sex was found for glutamate (females had a decrease in glutamate, males had an increase, comparing post- with pre-season).
Vedung (2022)	Sweden. *n* = 12 (50% female) athletes with repeated sports related concussion (rSRCs), compared to *n* = 11 (45% female) athlete controls. Control average age: 26, Concussed average age: 23	MRS – (NAA + NAAG)/Cr concentration ratios measured in grey and white matter. ASL measurements were taken across the brain to measure CBF changes.	Control group	One measurement completed based on athletes who had had a concussion 6 or more months ago and had persistent post-concussive symptoms	1. Widespread decrease of CBF in the female rSRC cohort compared to female healthy cohort; this was not found in the male rSRC cohort compared to male controls. Importantly, this change in CBF was not correlated with impaired cognitive function or symptom scores. 2. MRS did not find any sex differences in (NAA + NAAG)/Cr concentration ratios
Walter et al. (2023)	America. *n* = 12 (33% female) university athletes compared to *n* = 12 (50% female) athletes with no history of concussion. Control average age: 19.58, concussed average age: 19.75	MRI and ASL scans taken for four ROIs. MRI taken following the use of a head-cooling procedure as a possible neuroprotective therapeutic intervention.	Control Group	Measurements taken within the acute phase (< 10 days following injury)	No significant sex differences were found.
Wright, (2021)	Australia. *n* = 14 (43% female) concussed amateur Australian Rules players compared to *n* = 16 (44% female) amateur Australian Rules players without a recent history of concussion. Male average age: 23.4, Female average age: 24.3	dMRI taken, focusing on tracts in the corpus callosum, cingulum and corticospinal tract	Control group	48 h post-injury, 2 weeks post injury	1. Increased fibre density in cingulum of male athletes compared to females at both 48 h and 2 weeks following SRC 2. significant differences in fibre density and fibre cross-section, found in the corpus callosum and corticospinal tract.
Wright, (2022)	Australia. *n* = 13 (46% female) concussed amateur Australian Football athletes compared to *n* = 16 (43% female) control athletes without concussion. Male average age: 23.35, female average age: 24.23	MRI – 5 ROIs decided on throughout the brain. Used quantitative susceptibility mapping (QSM) to map cerebral venous oxygen saturation (SvO2)	Control group	Day 13 following concussion – day before they returned to play, according to regulation	Increased SvO2 in the straight sinus of concussed female athletes compared to control, whilst did not change in concussed male athletes.
Wu, (2018)	America. *n* = 27 (40% female) university athletes who had suffered a concussion compared to *n* = 27 (40% female) control university athletes. Concussed average age: 20.5, control average age: 21.5	Functional near-infrared spectroscopy (fNIRS) used to investigate oxygenated and deoxygenated haemoglobin in 6 ROIs across frontal and occipital regions whilst the participant completed sustained attention tasks.	Control group	Athletes tested at least 6 months after concussion occurred	No significant sex differences found.

*Note*. ASL, Arterial Spin Labelling; CBF, Cerebrospinal Fluid; CR, Creatine; dMRI, diffusion Magnetic Resonance Imaging; DTI, Diffusion Tensor Imaging; FA, Fractional Anisotropy; fMRI, functional Magnetic Resonance Imaging; fNIRS, Functional Near-Infrared Spectroscopy; MD, Mean Diffusion; MRI, Magnetic Resonance Imaging; MAP-MRI, Mean Apparent Propogator MRI; MRS, Metabolic Resonance Spectroscopy; NAA, N-acetylaspartate; QSM, Quantitative Susceptibility Mapping; RTP, Return to Play; ROI, Regions of Interest; SvO2, cerebral venous oxygen saturation

Fifteen studies were found to appropriately investigate sex differences following concussion with neuroimaging techniques. The neuroimaging techniques used were fMRI (*n* = 5), MRS (*n* = 3), ASL (*n* = 4) DTI (*n* = 2), QSM (*n* = 1), dMRI (*n* = 1), MAP-MRI (*n* = 1) and fNIRS (*n* = 1).

### MRS

MRS was used in three studies, with two studies using the ratio of NAA compared to Creatine as a reliable indicator of changes in neural structure (Chamard et al., 2012; Vedung, 2022), whilst the other measured NAA, glutamate, and choline concentrations (Panchal et al., 2018). Vedung et al. (2022) found no significant sex differences using MRS measurements of white and grey matter. Meanwhile, Chamard et al. (2012) did not find a difference between concussed males and females with NAA/Cr concentrations in the corpus callosum, but there was a significant decrease in these concentrations for the non-concussed female athlete population between the start and end of their seasons. Finally, Panchal et al. (2018) focused on the corpus callosum and showed a consistent decrease in NAA following concussion in both sexes. However, this study did suggest a trend level effect of sex difference in that glutamate decreased in females but increased in males. Interestingly, Panchal et al. did not use creatine as an energy marker, due to past research, which had identified it to change dramatically following mild injury (Yeo et al., 2011).

### Functional techniques

There were three studies that used fMRI and found no sex differences (Churchill et al., 2017, 2021a; Jarrett et al., 2016). Another study used fNIRS to identify cortical activation through haemoglobin levels in the brain whilst participants completed an attention task, finding no sex differences (Wu et al., 2018).

There were two studies that used fMRI and found sex differences (Churchill et al., 2020; Ly et al., 2022; Wright et al., 2022). Churchill et al. (2020) used resting-state fMRI and did not find a significant difference between males and females following concussion in functional connectivity. Ly et al. (2022) also utilised resting state fMRI to demonstrate lower functional connectivity involving visual, frontoparietal and default mode networks in concussed compared with non-concussed males, but no difference in functional connectivity between female groups.

### Quantitative susceptibility mapping (QSM)

Wright et al. (2022) used QSM and identified higher venous oxygen saturation in concussed compared to control females, a finding not evident in the male concussed and control populations.

### Arterial spin labelling (ASL)

ASL can measure cerebral blood flow (CBF) by using labelled arterial blood water to measure brain function with regard to oxygen supply, providing an indication of cognitive functioning (Wang et al., 2016). It was used in four studies, with Vedung et al. (2022) studying symptomatic athletes with multiple concussions and identifying a decrease across all regions of the brain in CBF in concussed compared to non-concussed females, which was not found in the male samples. However, two studies found concussion to impact CBF more profoundly in males compared to females. Hamer et al. (2020) tested previously concussed asymptomatic athletes and identified a decrease in CBF in male athletes compared to males without a history of concussion. Conversely, female athletes with a history of concussion demonstrated a large variance in CBF and did not demonstrate this difference in CBF compared to females without a history of concussion.

Further analyses identified a decrease in CBF in females who had experienced multiple previous concussions compared to females who had experienced only one concussion, though this pattern was not evident in males. Churchill et al. (2021b) tracked athletes from the acute phase for an extended period, identifying a greater reduction in occipital-parietal CBF in males compared to females, with the greatest effect being found 1-year post-RTP. Interestingly, the male and female athletes did not present with any differences in symptomology post-concussion, nor did they differ in time to attain medical clearance for return to play. Walter et al. (2023) attempted to use head cooling as a treatment to reduce CBF changes but did not find any significant sex differences.

### Diffusion techniques

Diffusion was observed in four studies, with two studies finding a larger impact following concussion on females. Churchill et al. (2021b) investigated diffusion and white matter integrity and found these to be more impaired for concussed females, particularly in the corpus callosum and posterior thalamic radiation when compared to concussed males. Ly et al. (2022) did not find any sex differences in diffusion measures, however in this study concussion group was not found to significantly predict any DTI measures. Wright et al. (2021) used diffusion MRI to analyse the corpus callosum and corticospinal tract, where there are fibre bundles crossing. They found that males had more fibre density than females 2 weeks following concussion compared to 48 h post-injury, suggesting males suffered more structural damage than females following concussion. Goeckner et al. (2024) used MAP-MRI, a novel and sensitive measure of nonparametric diffusion, finding widespread white matter microstructural damage was associated with a history of concussion, while this association was much stronger in females compared to male athletes. Exploratory grey matter analyses also revealed sex differences in the regions affected in athletes with a history of concussion (Goeckner et al., 2024). For example, MAP-MRI analyses showed abnormalities in bilateral thalamus for female athletes with a history of concussion, while male athletes showed abnormalities in bilateral pallidum and putamen.

## Discussion

The aim of this systematic review was to evaluate the neuroimaging literature that addressed sex differences in sports-related concussion. This identified 15 studies, with the majority reporting a difference between males and females in their concussion recovery. Despite this broad conclusion, the nature of these differences was highly variable, with some studies finding more change and others finding less change in female brain structure or function compared with males following concussion. This reflects the lack of comprehensive research that has used neuroimaging to investigate sex differences in sports-related concussion and highlights the need for more systematic research into any potential differences in recovery time.

Technologies such as MRS investigated metabolic differences, and either found no sex difference (Vedung et al., 2022; Chamard et al., 2012) or a trend level effect of sex with females having an increase, but males a decrease in glutamate over a season (Panchal et al., 2018). Any interpretation based on these mixed findings must be cautious, however. For example, Chamard et al. (2012) did not find significant differences between concussed male and female athletes, but interestingly there was a significant decrease in NAA/Cr concentration in non-concussed female athletes which was not identified in non-concussed male athletes. This was suggested to be due to sub-concussive knocks having impacted females more, with lower NAA/Cr concentrations in females compared to males. Studies that failed to find significant differences with MRS suggested that whole brain scans did not identify focal issues, likely due to the statistical corrections required for such a large quantity of comparisons (Vedung et al., 2022). Future studies with larger sample sizes could offer greater statistical power to draw reliable conclusions regarding sex differences in metabolism.

Different metabolites underlined varying changes across sex following sports-related concussion. Panchal et al. (2018) were the only study to measure glutamate, a metabolite important for excitatory neurotransmission. Although the overall interaction between time (pre- vs. post-season) and sex (*p* =.03) did not survive correction for comparisons of multiple metabolites, the authors found a slight decrease in females sustaining repeated head impacts over a single season, whereas male athletes demonstrated an increase in glutamate over the season.

Immediately following a biomechanical brain injury, there is diffuse axonal injury and damage to neuronal membranes leading to a complex neurometabolic cascade. This includes increased influx of calcium and subsequently indiscriminate release of glutamate that bind to post-synaptic N-methyl-D-aspartate (NMDA) receptors, resulting in further cell depolarization (Anderson et al., 2006; Blennow et al., 2012; Giza & Hovda, 2001). This cascade can lead to glutamate excitotoxicity and potential apoptosis (Panchal et al., 2018). However, female sex hormones like estrogen regulate glutamate changes to avoid glutamate excitotoxicity (Mize et al., 2003). These sex hormones that are much higher in females than males may significantly impact the metabolic reaction following concussion and could cause a major point of difference in response to injury (La Fountaine et al., 2019). Estrogen has a facilitating effect on glutamate transmission (Yokomaku et al., 2003), potentially regulating glutamate levels and reducing excitotoxicity in females, though this effect may be less pronounced in males. This sex difference could explain the different glutamate reactions found by Panchal et al. (2018). Their study also demonstrated a negative correlation between change in glutamate and verbal memory, suggesting symptomatic consequences of these metabolite changes. Excess glutamate may over-activate NMDA receptors, resulting in excitotoxicity and interrupting brain function (Hynd et al., 2004). The corpus callosum, which was the focus for Panchal et al. (2018), is crucial for language processing, indicating that glutamate excitotoxicity may impact the ability to store verbal information. This result suggests that more research into metabolic feedback following concussion could be beneficial in understanding how males and females differ in recovery, particularly in understudied metabolites like glutamate. Additionally, more investigation of the impact of sex hormones in concussion recovery for females could highlight important differences from males, altering the appropriate point of return to play for each gender.

Functional techniques and ASL were employed to explore functional differences between male and female athletes following concussion. Resting-state fMRI was employed by Churchill et al. (2020) to examine the temporal dynamics of the BOLD response across a range of time scales in healthy and concussed athletes. It identified differences in temporal dynamics in prefrontal, occipital and subcortical regions between male and female athletes, but concussion did not interact with this sex (Churchill et al., 2020). Meanwhile, when compared with non-concussed male athletes, concussed males showed reduced resting-state functional connectivity between visual and frontoparietal networks, and between visual and default mode networks, but this effect was not found in female athletes (Ly et al., 2022). This could suggest that males’ ability to process visual information or allocate visual attention following concussion could be decreased, putting themselves in danger of re-injury with impacted processing abilities of their environment (Shah-Basak et al., 2018). This appears to contrast with an earlier study in a sample of concussed females demonstrating a significant reduction in structural integrity in the motor networks at least 7 months post-concussion in comparison to non-concussed females (Chamard et al., 2013).

Other functional studies also identified differences between males and females following concussion by measuring labelled arterial blood water and diffusion within the brain (Vedung et al., 2022; Churchill et al., 2021b). Wright et al. (2021) used dMRI to find a more significant impact in fibre density with males compared to females two weeks post-injury. Interestingly, a factor that was identified as contributing to concussion and recovery for males was the time spent playing sport, which for males was typically almost double that of female athletes in each contact sport (Wright et al., 2021). This increased contact time may have caused more sub-concussive or concussive impacts, which could negatively impact their long-term brain functionality and recovery (Mainwaring et al., 2018). Furthermore, the functional differences between males and females may be due to estrogen and progesterone having a neuroprotective influence on females, which as previously mentioned fluctuates throughout the menstrual cycle (Gallagher et al., 2018). Hamer et al. (2020), despite only finding lower CBF in male but not female concussed athletes compared to controls, did find that there was a higher variance in the female concussion group. If sex hormones do have an impact on concussion recovery, the reported greater variation could be due to the fact that females may be at different stages of their menstrual cycle. These are important factors that may differentiate females and males in their recovery from concussion, despite not being accounted for in the reviewed studies.

A further contributor to neuroprotective effects of sex hormones relates to the greater cerebrovascular reactivity reported in women (Krause et al., 2006). Previous studies have identified healthy females to have a stronger vasodilatory response to changes in carbon dioxide pressure (Kastrup et al., 1997), while hormones have previously been linked to cerebral blood flow (Armstead, 2016). Together these findings suggest that females may buffer the concussion-induced changes that occur in blood flow (Churchill et al., 2021b) and this could potentially impact the reliability of BOLD measurements (Hamer et al., 2020). Considering these functional findings in conjunction with the reported metabolic sex differences in glutamate levels (Panchal et al., 2018) underscores the relevance of sex and its impact on the response to concussion.

Structural sex differences were highlighted by diffusion techniques. Two studies suggested concussed females were impacted more in important white matter structures compared to males (Churchill et al., 2021b; Goeckner et al., 2024). However, another structural study suggested no sex differences in DTI measures (Ly et al., 2022). These studies differ in the timing of patient testing, with some testing patients within 72 h of injury (Ly et al., 2022) while others focussed on post-acute effects 6–12 months after injury (Churchill et al., 2021b; Goeckner et al., 2024). Females being more prone to significant structural injury may be explained by differences in head-neck mass. For example, if a player hits the ground following a tackle, reduced head-neck mass provides less support and greater biomechanical forces can be transmitted to the brain (Tierney et al., 2008). A further contributing factor comes from in vitro evidence that female axons are smaller than male axons and at greater risk of damage following trauma (Dollé et al., 2018). Further examination of where most of the structural brain damage that occurs following a concussion, and how it differentiates between sex, is important to account for regarding regions of vulnerability for specific athletes.

## Conclusions

Overall, this systematic review sought to identify any differences between male and female athletes in sports-related concussion and the recovery process following it. It is clear there needs to be a much greater focus on this question, and definitive conclusions are not possible based on the existing evidence. Nevertheless, although not consistently established, many studies reported greater neurobiological impact of concussion in female athletes, which is consistent with previous reviews of symptom report and cognitive assessment (Covassin & Elbin, 2011; Broshek et al., 2005). The findings presented should act as an incentive for future research to continue the investigation of sex differences following concussions in athletes, and if recovery protocols should differ for female and male athletes.

Despite these studies offering valuable findings, they were not without their limitations. A widespread issue, particularly for studies that did not report sex differences, was sample size. Notably, of the 15 studies included in the current review, only five were specifically designed with the aim to test sex differences. Some studies included in our review involved concussion samples with as low as 4 females and hence were not likely to be well powered for testing for sex differences. Furthermore, the effect of past concussions or sub-concussive impacts on these studies may play a role, but was not typically accounted for, and warrants further investigation. Churchill et al. (2021b) for example included a sample of control athletes with a history of concussion, whilst Hamer et al. (2020) simply asked individuals to self-report their past concussions, which is prone to incorrect reporting. Past research has highlighted the effect of concussion history on structural and functional integrity, as well as the impact of sub concussive injuries (Sollmann et al., 2018). In future work, control participants from outside athletic backgrounds and without a concussion history may be more suitable. Utilising individuals who deal with pain and similar sociological problems like stress following injury – for example orthopedic patients – may offer a more appropriate control group controlling for nonspecific effects of traumatic injury (Wilde et al., 2019).

Additionally, there were some limitations within the current review process. Paediatric studies were excluded since children have been shown to respond to concussion differently (Davis et al., 2017). Future studies would benefit from understanding the physiological impact of concussion and any sex differences that may be evident for minors, given that concussed female adolescents in previous studies were shown to suffer worse symptoms than concussed adolescent males following sports-related concussion (Berz et al., 2013; Tanveer et al., 2017). This would provide more appropriate recovery protocols and ensure better protection of the developing brain of children when participating in contact sport. Furthermore, this review focused only on sports-related concussion. Existing research suggests that mechanism of injury may be relevant, with greater symptoms reported despite similar severity of injury and neurocognitive function (Shumski et al., 2023; Tarkenton et al., 2021). While most studies have focussed on sports-related concussion, there is little research directly comparing brain changes across different mechanisms of injury (such as motor vehicle accidents, assaults etc.). Future research should investigate what impact mechanism of injury have on the recovery period following concussion using neuroimaging techniques.

This study has highlighted some differences between males and females following sports-related concussion, but more research is needed to understand how they differ and why. Such a conclusion parallels those established in a scoping review into sex differences in the broader literature on TBI (Gupte et al., 2019). Evidence that patients with no clinical observations nevertheless demonstrate neurological impairment (Churchill et al., 2021b) points to the limitations of current gold-standard measures for monitoring concussion (e.g., SCAT6). More research into cost-effective strategies (e.g., functional near infrared spectroscopy; Hocke et al., 2018) to track concussion recovery could be useful for more accurate diagnosis and tracking recovery. Techniques like eye-tracking have been shown to not only effectively reflect concussion in individuals but has also differentiated males and females to show that females are more affected in oculomotor and vestibular areas (Jain et al., 2022). In many respects, research into physiological underpinnings of concussion is still in its infancy, while a comprehensive understanding of the impact on male and female patients is in urgent need of further research. This endeavour has important implications for guiding sports rules and concussion protocols to keep athletes safe in the long-term.

## Electronic supplementary material

Below is the link to the electronic supplementary material.

Supplementary Material 1

## Data Availability

No datasets were generated or analysed during the current study.
