# Supplementary material for: Using neuroimaging to identify sex differences in adults with sports-related concussion: a systematic review
Source: Brain Imaging Behav. 2025 Jan 24;19(2):594–608. doi: 10.1007/s11682-025-00970-6 (PMC11978690; doi:10.1007/s11682-025-00970-6)
Supplement: Supplementary file 1 — Supplementary Material 1 [file 11682_2025_970_MOESM1_ESM.docx]

**Supplementary Table 1. Modified Downs and Black checklist for assessment of methodological quality.**

|  | Chamard (2012) | Churchill (2017) | Churchill (2021a) | Churchill (2021b) | Churchill (2020) | Goeckner (2023) | Hamer (2020) | Jarrett (2016) |
| --- | --- | --- | --- | --- | --- | --- | --- | --- |
| Q1 Hypothesis clearly described | 1 | 1 | 1 | 1 | 1 | 1 | 1 | 0 |
| Q2 Main outcomes in introduction or methods | 1 | 1 | 1 | 1 | 1 | 1 | 1 | 0 |
| Q3 Patient characteristics clearly described | 1 | 1 | 1 | 1 | 1 | 1 | 1 | 1 |
| Q4 Interventions of interest clearly described | 1 | 1 | 1 | 1 | 1 | 1 | 1 | 1 |
| Q5 Principal confounders clearly described | 1 | 1 | 1 | 1 | 1 | 1 | 0 | 1 |
| Q6 Main findings clearly described | 1 | 1 | 1 | 1 | 1 | 1 | 1 | 1 |
| Q7 Estimates of random variability provided for main outcomes | 0 | 1 | 1 | 0 | 1 | 0 | 0 | 0 |
| Q8 All adverse events of intervention reported | 1 | 0 | 1 | 0 | 1 | 0 | 0 | 1 |
| Q9 Characteristics of patients lost to follow-up described | 0 | 0 | 1 | 1 | 1 | 1 | 0 | 1 |
| Q10 Probability values reported for main outcomes | 0 | 0 | 1 | 1 | 1 | 1 | 1 | 1 |
| Q11 Subjects asked to participate were representative of source population | 1 | 1 | 1 | 1 | 1 | 1 | 1 | 1 |
| Q12 Location and delivery of study treatment was representative of source population | 1 | 1 | 1 | 1 | 1 | 1 | 1 | 1 |
| Q13 Study participants blinded to treatment | UTD | UTD | UTD | UTD | UTD | UTD | UTD | UTD |
| Q14 Blinded outcome assessment | 1 | 1 | 1 | 1 | 1 | 1 | 1 | 1 |
| Q16 Any data dredging clearly described | 1 | 1 | 1 | 0 | 1 | 0 | 0 | 1 |
| Q17 Analyses adjust for differing lengths of follow-up | 1 | 0 | 0 | 0 | 1 | 1 | 0 | 0 |
| Q18 Appropriate statistical tests performed | 1 | 1 | 1 | 1 | 1 | 1 | 1 | 1 |
| Q19 Compliance with interventions was reliable | 1 | 1 | 1 | 0 | 1 | 1 | 1 | 1 |
| Q20 Outcome measures were reliable and valid | 1 | 1 | 1 | 1 | 1 | 1 | 1 | 1 |
| Q21 All participants recruited from same source population | 1 | 1 | 1 | 1 | 1 | 1 | 1 | 1 |
| Q22 All participants recruited over same time period | 1 | 1 | 1 | 1 | 1 | 1 | 1 | 1 |
| Q23 Participants randomised to treatment | UTD | UTD | UTD | UTD | UTD | UTD | UTD | UTD |
| Q24 Allocation of treatment concealed from investigators and participants | UTD | UTD | UTD | UTD | UTD | UTD | UTD | UTD |
| Q25 Adequate adjustment for confounding | 0 | 1 | 0 | 1 | 0 | 0 | 0 | 0 |
| Q26 Losses to follow-up taken into account | 0 | 1 | 1 | 0 | 0 | 0 | 0 | 0 |
| Sufficient power to detect treatment effect at significance level of 0.05 | 1 | 0 | 1 | 1 | 1 | 1 | 1 | 1 |
| Total | 19 | 19 | 22 | 18 | 22 | 18 | 16 | 17 |

*Note*. UTD, unable to determine.

|  | Ly (2022) | Panchal (2018) | Vedung (2022) | Walter (2023) | Wright (2021) | Wright (2022) | Wu (2018) |
| --- | --- | --- | --- | --- | --- | --- | --- |
| Q1 Hypothesis clearly described | 1 | 1 | 1 | 1 | 1 | 0 | 1 |
| Q2 Main outcomes in introduction or methods | 1 | 1 | 1 | 1 | 1 | 1 | 1 |
| Q3 Patient characteristics clearly described | 0 | 1 | 1 | 1 | 1 | 1 | 1 |
| Q4 Interventions of interest clearly described | 1 | 1 | 1 | 1 | 1 | 1 | 1 |
| Q5 Principal confounders clearly described | 1 | 0 | 0 | 1 | 1 | 1 | 1 |
| Q6 Main findings clearly described | 1 | 1 | 1 | 1 | 1 | 1 | 1 |
| Q7 Estimates of random variability provided for main outcomes | 0 | 1 | 1 | 0 | 0 | 0 | 0 |
| Q8 All adverse events of intervention reported | 1 | 1 | 1 | 1 | 1 | 1 | 1 |
| Q9 Characteristics of patients lost to follow-up described | 1 | 1 | 1 | 0 | 1 | 1 | 1 |
| Q10 Probability values reported for main outcomes | 1 | 0 | 1 | 1 | 1 | 1 | 1 |
| Q11 Subjects asked to participate were representative of source population | 1 | 1 | 0 | 1 | 1 | 1 | 1 |
| Q12 Location and delivery of study treatment was representative of source population | 0 | 1 | 1 | 1 | 1 | 1 | 1 |
| Q13 Study participants blinded to treatment | UTD | UTD | UTD | 0 | UTD | UTD | UTD |
| Q14 Blinded outcome assessment | 0 | 1 | 1 | 1 | 1 | 1 | 1 |
| Q16 Any data dredging clearly described | 0 | 1 | 1 | 1 | 1 | 1 | 1 |
| Q17 Analyses adjust for differing lengths of follow-up | 1 | 1 | 0 | 0 | 1 | 1 | 1 |
| Q18 Appropriate statistical tests performed | 1 | 1 | 1 | 1 | 1 | 1 | 1 |
| Q19 Compliance with interventions was reliable | 1 | 1 | 1 | 1 | 1 | 1 | 1 |
| Q20 Outcome measures were reliable and valid | 1 | 1 | 1 | 1 | 1 | 1 | 1 |
| Q21 All participants recruited from same source population | 0 | 1 | 0 | 1 | 1 | 1 | 1 |
| Q22 All participants recruited over same time period | 1 | 1 | 1 | 1 | 1 | 1 | 1 |
| Q23 Participants randomised to treatment | UTD | UTD | UTD | 1 | UTD | UTD | UTD |
| Q24 Allocation of treatment concealed from investigators and participants | UTD | UTD | UTD | 0 | UTD | UTD | UTD |
| Q25 Adequate adjustment for confounding | 0 | 1 | 0 | 1 | 0 | 0 | 0 |
| Q26 Losses to follow-up taken into account | 0 | 1 | 1 | 0 | 0 | 0 | 1 |
| Sufficient power to detect treatment effect at significance level of 0.05 | 1 | 1 | 1 | 1 | 1 | 1 | 1 |
| Total | 15 | 21 | 18 | 20 | 20 | 19 | 21 |

*Note*. UTD, unable to determine.
